# MicroRNAs at the human 14q32 locus have prognostic significance in osteosarcoma

**DOI:** 10.1186/1750-1172-8-7

**Published:** 2013-01-11

**Authors:** Aaron L Sarver, Venugopal Thayanithy, Milcah C Scott, Anne-Marie Cleton-Jansen, Pancras CW Hogendoorn, Jaime F Modiano, Subbaya Subramanian

**Affiliations:** 1 Masonic Cancer Center, University of Minnesota, 11-212 Moos Tower , 515 Delaware Street S.E, Minneapolis, MN 55455, USA; 2 Department of Surgery, Division of Basic and Translational Research, School of Medicine, University of Minnesota, Minneapolis, MN 55455, USA; 3 Department of Veterinary Clinical Sciences, College of Veterinary Medicine, University of Minnesota, St. Paul, MN, 55108, USA; 4 Department of Pathology, Leiden University Medical Center, Leiden, 2333ZA, The Netherlands

**Keywords:** Bone neoplasm, Prognosis, Integrative analysis, Osteosarcoma, 14q32 miRNAs

## Abstract

**Background:**

Deregulation of microRNA (miRNA) transcript levels has been observed in many types of tumors including osteosarcoma. Molecular pathways regulated by differentially expressed miRNAs may contribute to the heterogeneous tumor behaviors observed in naturally occurring cancers. Thus, tumor-associated miRNA expression may provide informative biomarkers for disease outcome and metastatic potential in osteosarcoma patients. We showed previously that clusters of miRNAs at the 14q32 locus are downregulated in human osteosarcoma.

**Methods:**

Human and canine osteosarcoma patient’s samples with clinical follow-up data were used in this study. We used bioinformatics and comparative genomics approaches to identify miRNA based prognostic biomarkers in osteosarcoma. Kaplan-Meier survival curves and Whitney Mann U tests were conducted for validating the statistical significance.

**Results:**

Here we show that an inverse correlation exists between aggressive tumor behavior (increased metastatic potential and accelerated time to death) and the residual expression of 14q32 miRNAs (using miR-382 as a representative of 14q32 miRNAs) in a series of clinically annotated samples from human osteosarcoma patients. We also show a comparable decrease in expression of orthologous 14q32 miRNAs in canine osteosarcoma samples, with conservation of the inverse correlation between aggressive behavior and expression of orthologous miRNA miR-134 and miR-544.

**Conclusions:**

We conclude that downregulation of 14q32 miRNA expression is an evolutionarily conserved mechanism that contributes to the biological behavior of osteosarcoma, and that quantification of representative transcripts from this family, such as miR-382, miR-134, and miR-544, provide prognostic and predictive markers that can assist in the management of patients with this disease.

## Background

Osteosarcoma is the most common primary bone tumor and predominantly affects children and adolescents [1,2]. The impact of osteosarcoma on patients, families, caregivers, and the extended community (*e.g.*, the sociological impact on their peers) is disproportionate to the numbers affected both due to morbidity and years of life lost. Due to the infrequent occurrence as well as the heterogeneous nature of osteosarcoma in humans, the molecular understanding of this disease has progressed slowly as compared to other cancer types. However, the occurrence of an orthologous disease in dogs provides an opportunity to surmount some of these challenges.

In contrast to humans, osteosarcoma is relatively common in dogs (estimated at >10,000 cases per year), with exquisite breed predilection. Osteosarcoma arises spontaneously in dogs, and its biology is similar to the human disease both anatomically and functionally [3]. The chronology of the disease is adapted to lifetime, so progression of osteosarcoma in dogs takes place in a time frame (~2 years) that allows rapid assessment of risk, prediction, and outcome [3]. The availability of detailed genome maps for humans and dogs, and the extensive homology in sequence and gene order between both species makes integration and extrapolation of genomic data feasible [4,5].

We have previously identified sets of differentially expressed genes that are associated with tumor behavior in both canine and human osteosarcoma [6]. Specifically, we characterized gene signatures that can stratify canine and human osteosarcoma according to the predicted biological behavior of the tumors [6]. The signatures are characterized by increased cell cycle gene expression that regulate G2/M transition and DNA damage genes in dogs with worst outcomes. They are typified by increased expression of ‘microenvironment interactions’ genes in dogs with better outcomes. These patterns also were observed in human osteosarcoma tumor datasets, but the mechanisms responsible for generating these signatures have not yet been fully defined.

MicroRNAs have potential uses in diagnosis and prognosis of tumors [7,8]. In our previous work, we used miRNA profiles from over 300 human sarcoma samples included in our S-MED database http://www.oncomir.umn.edu[9] to identify unique miRNA expression profiles for osteosarcoma. Specifically, our data showed highly significant downregulation of a large number of miRNAs at the 14q32 locus in human osteosarcoma compared to normal bone tissue, osteoblasts and other types of sarcomas [10]. In this study, we demonstrate an inverse correlation between the residual expression of 14q32 miRNA expression and aggressive biological behavior in osteosarcoma from humans and dogs.

## Materials and methods

### Human osteosarcoma tissue samples

Human tissues were obtained from two sources. Frozen osteosarcoma tissue samples and normal bone samples (femur/ tibia) of individuals from similar age groups were obtained through the tissue procurement facility at the University of Minnesota (Bionet). Demographic and clinical information is provided in Additional file 1: Table S1 and Additional file 2: Table S2. The excised primary osteosarcoma tumors were obtained prior to the initiation of chemotherapy or radiotherapy with informed consent and following institutional review board-approved protocols. Surgical resections were subjected to standard clinical and histopathological evaluation of hematoxylin and eosin (H&E) stained sections. An additional 16 human osteosarcoma samples with complete clinical follow-up information, including outcomes, were obtained from Pancras C.W. Hogendoorn with approval from the institutional review board in compliance with the declaration of Helsinki. Demographic and clinical information is provided for these samples in Additional file 3: Table S3.

### Canine osteosarcoma tissue samples and cell lines

The procedures to obtain canine osteosarcoma samples and establish corresponding explants and canine cell lines have been described previously [6,11]. Briefly, naïve (prior to treatment) samples were obtained from family-owned pet dogs with stage I or stage II appendicular osteosarcoma. Thirty-four of the 37 canine osteosarcoma samples used in this study were derived from the primary tumor in the leg. Samples OSCA-6 (Osteosarcoma Cells ACCR-6), OSCA-11, and OSCA-16 were derived from pulmonary metastases. Participation in the study required the dogs’ owners to sign an informed consent form indicating they understood the goals and procedures for this study. Protocols and procedures were reviewed by the appropriate Institutional Animal Care and Use Committee. Samples were collected by attending veterinarians under sterile conditions as part of a diagnostic biopsy procedure, immediately after limb amputation, or at necropsy. Grossly visible tumor was dissected from adjacent normal tissue, rinsed in sterile phosphate buffered saline solution and divided into three portions that were (1) fixed in 10% neutral buffered formalin, (2) snap frozen, and (3) disaggregated into single cell suspensions. Histologically, the cellular composition of the resulting trimmed samples consisted of >90% tumor cells. For the single cell suspensions, debris was removed by passing sequentially through 500- and 200-μm mesh filters. Cells were then placed in a 4 cm^2^ culture dish at a density of 1 x 10^6^/ml in 1 ml of DMEM supplemented with 10% fetal bovine serum and cultured at 37°C in a 5% CO_2_ atmosphere. Cells were allowed to reach ~80% confluence, at which time they were passed to a 10-cm^2^ culture dish, and then again to a 25-cm^2^ culture flask. By the second passage, the cultures were homogeneous and consisted of large polygonal to plump spindloyd or slightly rounded cells. Histologic diagnosis of osteosarcoma was verified for all tumors from formalin-fixed tissues. Osteoblastic origin of the cultured cells was confirmed at the third passage by expression of alkaline phosphatase and osteocalcin. As needed, cells were maintained in culture using the same conditions described above. Selected cell lines were tested after 30 passages in culture and had no noticeable changes in morphology or loss of alkaline phosphatase expression. These cell lines are tumorigenic *in vivo*, and, when injected orthotopically into nude mice, they have the capacity to metastasize spontaneously to the lungs [12]. Demographic and clinical information for the canine samples is provided in Additional file 4: Table S4.

### RNA isolation

Total RNA was isolated from 75–100 mg of frozen human osteosarcoma tissue using the miRvana total RNA isolation kit (Ambion Inc, Austin TX, USA) following the manufacturer’s protocol. RNA was quantified using the Nanodrop 8000 (NanoDrop Technologies LLC, Wilmington, DE, USA). Samples with RNA Index Number (RIN) values of >6 were included in this study. Total RNA was isolated from canine osteosarcoma tissue using TriPure Isolation Reagent (Roche, Germany). The concentration of RNA from canine osteosarcoma tissue was determined using NanoDrop 1000 UV–vis spectrophotometer (NanoDrop Technologies) and quality was assessed on a 1.2% formaldehyde agarose gel with ethidium bromide staining. RNA from canine osteosarcoma cryopreserved cells was isolated, quantified, and assessed for quality as previously described [6].

### Analysis of osteosarcoma mRNA and miRNA expression

Messenger RNA expression profiles from human tumors were generated using the human HT-12 Beadchip (Illumina Inc., San Diego, CA, USA) [13]. Human miRNA expression profile datasets described previously (S-MED)[9] were used in this study. Additional clinical information relating to these samples is described in Additional file 1: Tables S1 (human). Canine osteosarcoma mRNA gene expression data generated on Affymetrix canine_2.0 arrays were also used as previously described [6]. Complete clinical and breed information for canine osteosarcoma data is provided in Additional file 4: Table S4. miRNAs from the 14q32 cluster were mapped to the canine genome. Two miRNAs (miR-134 and miR-544) that showed 100% conservation and mapped to the predicted region of synteny in canine chromosome (CFA) 8 were used to examine expression of the 14q32 cluster in dog samples (Additional file 5: Figure S1).

**Figure 1:**
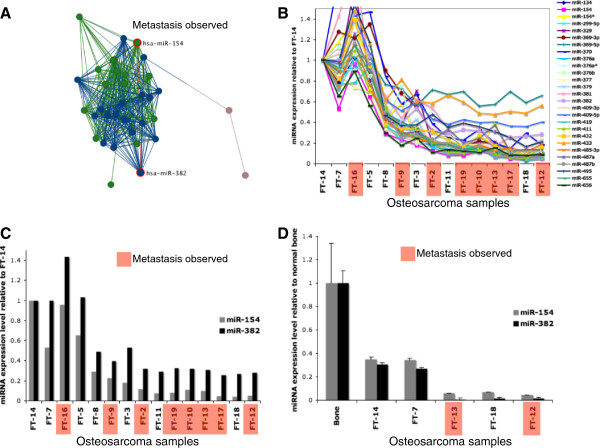
**14q32 miRNA expression levels and metastasis in human osteosarcoma.** (**A**) Correlation network of 14q32 miRNAs expression in human osteosarcoma samples. Correlation network was constructed with Pearson correlation R >0.9 as the edges. The resulting correlation sub-network contained thirty-six miRNAs and 33 of them mapped to the 14q32 locus. All pairwise correlations used to generate this image are given in Additional file 8: Table S7. (**B**) Microarray based transcript levels of highly correlated 14q32 miRNAs in osteosarcoma tumors shown relative to the level observed in FT-14 (human osteosarcoma primary tumor). miRNA profiling data from human osteosarcoma tumors where metastasis was observed show stronger downregulation of 14q32 miRNAs (highlighted in red) than primary osteosarcoma tumors where metastasis was not observed. (**C**) Microarray based transcript levels for miR-382 and miR-154. miR-382 and miR-154 show a high level of correlation (R^2^ = 0.95). (**D**) qRT-PCR validation for miR-382 and miR-154 expression levels obtained in primary osteosarcoma tumors (FT-14, -7) where metastasis was not observed and three primary osteosarcoma tumors with low levels of (FT-18, -13, -12) where metastasis was observed. miR-382 and miR-154 show a high level of correlation (R^2^ = 0.99). Measurements were carried out in triplicate and were normalized to the expression levels in two independent normal bone tissues that were also carried out in triplicate.

For human samples, mRNA data were assayed for quality control as described [14]. Fluorescence values were obtained from the Illumina detection system without background subtraction and were quantile normalized using GeneData Expressionist Software (GeneData Inc, San Francisco, CA, USA). Multiple probes to the same gene product were then averaged to obtain a value for each gene. The dataset was filtered to identify genes with a variance >1 across the human osteosarcoma tumor dataset and two normal bone samples. The mRNA expression data were composed of human tumors and normal bone samples which had been previously profiled for miRNA expression levels, allowing us to directly calculate Pearson correlation coefficients between miR-382 and mRNA transcript levels. mRNAs that showed positive correlation > 0.7 are listed in Additional file 6: Table S5, and mRNAs that showed negative correlation are listed < −0.7 in Additional file 7: Table S6. These mRNAs and their direction of change (increased for negative correlation and decreased for positive correlation) were submitted to Ingenuity Pathway Analyses to determine enriched functions where the direction of change was consistent with increased or decreased activity of enriched functions. The quantile normalized human miRNA array data were first filtered to determine miRNAs that showed variance >0.1 across the 15 human osteosarcoma tumors. Pearson correlations were then calculated between all pairs of miRNA. All pairs of miRNA with R^2^ >0.9 were then used to generate a correlation network visualization of the osteosarcoma miRNAome. Pairwise Pearson correlations used to generate Figure 1A are provided in additional file 8: Table S7.

### Integrative analysis of human and canine gene and miRNA expression data

Human genes that showed positive and negative correlation to miR-382 were mapped to canine genes using gene symbols. The direction of change was tracked using blue (negative correlation) and yellow (positive correlation) tags for each gene so that the direction of change in the human data could be observed in analogous fashion as previously described osteosarcoma [6]. Unsupervised hierarchical clustering was carried out with the Pearson correlation as the metric using average linkage following log base 2 transformation of mean-centered data using Cluster3.0. Heatmaps were generated from the CDT files generated by Cluster3.0 using Java Treeview [15].

### qRT-PCR

qRT-PCR of RNA from both human and canine osteosarcoma samples was carried out using normalization to U6 snRNA. First strand cDNA was synthesized from total RNA using a miScript reverse transcription kit (Qiagen, Valencia, CA, USA). miRNA was quantified with an miRscript SYBR Green PCR kit (Qiagen) using cDNA equivalent of 50 ng total RNA per reaction. Mature miRNA-specific forward primers provided in Table 1 were purchased from Integrated DNA Technologies (Coralville, IA, USA) and the universal reverse primer provided by the manufacturer. Real-time PCR was performed at 55°C annealing following standard protocol of the manufacturer in 7500 Real Time PCR system (Applied Biosystems/Life Technologies, Inc., Carlsbad, CA USA) and threshold cycles (CT) were calculated using Sequence Detection Software (SDS v1.2.1, Applied Biosystems). Relative gene expression (expressed as fold difference relative to normal bone tissues (human osteosarcoma) or relative to reactive osteoblasts (canine osteosarcoma)) was calculated from the average of triplicate CT value measurements using the 2-ΔΔCt method [16] except for situations where the RNA available was limited allowing only duplicate reactions to be carried out. The standard error is represented as 2^-(ΔΔCT ± SD)^.

**Table 1:** Oligos used for qRT-PCR analysis

**Name and miRbase number of the microRNA**	**Oligo sequence**
miR-154 MIMAT0000452	GGAGGTTATCCGTGTTGCCTTCG
miR-382 MIMAT0000737	GGGGAAGTTGTTCGTGGTGGATTCG
miR-544 MIMAT0003164	GGGGGATTCTGCATTTTTAGCAAGTTC
miR-134 MIMAT0000447	TGTGACTGGTTGACCAGAGGGG

### Statistical analyses

Kaplan-Meier survival curves were generated with the GeneData Analyst statistical package using the clinical data provided in Additional file 3: Table S3 and Additional file 4: Table S4. Whitney Mann U tests were conducted using an online tool available at http://vassarstats.net/utest.html.

## Results

### 14q32 miRNAs expression level decreases are highly correlated in human osteosarcoma

Over 40 miRNAs are found to be present between the imprinted genes *DLK1* and *DIO3* at the human 14q32 locus [17]. We previously showed that these miRNA are significantly downregulated in human osteosarcoma compared to normal bone tissues [10]. We also showed that these miRNA are downregulated in human osteosarcoma relative to other types of sarcomas [9]. In order to verify that the 14q32 miRNA decreases in expression levels are highly correlated in each of the individual tumor tissue samples, we generated a correlation-based network using the miRNA expression levels determined by array analyses. The analysis revealed that miRNA expression levels found at the 14q32 locus were highly correlated (Figure 1A and Additional file 8: Table S7). The high correlation in expression levels suggests that an individual miRNA from this locus could represent the expression levels of most of 14q32 miRNAs in osteosarcoma.

### Magnitude of 14q32 miRNA decreases in human osteosarcoma vary significantly

To demonstrate that the high level of correlation observed for 14q32 miRNAs was derived from meaningful decreases in expression levels, we plotted the expression levels of the 14q32 miRNA members in human osteosarcoma relative to the human osteosarcoma primary tumor sample FT-14. FT-14 showed a relatively high level of 14q32 miRNAs (Figure 1B). Several of the osteosarcoma tumor samples showed 14q32 miRNA levels similar to those observed in FT-14 while several of the other osteosarcoma tumors showed much more sizable decreases in 14q32 miRNAs. The magnitude of miRNA expression decrease ranged from ~3-fold to ~20-fold relative to the levels observed in normal bone tissue by miRNA microarray analyses [9,10]. The heterogeneity in expression levels was seen in 14q32 locus miRNAs, and is specifically shown for two members at this locus: miR-382 and miR-154 (Figure 1C). The expression levels observed for miR-382 and miR-154 showed a high level of correlation (R^2^ = 0.95), exemplary of the high correlations observed across the 14q32 miRNAs. In order to confirm that the results obtained by miRNA microarray for the magnitude of the 14q32 miRNA decreases were meaningful, we carried out qRT-PCR for miR-382 and miR-154. The results show that, relative to normal bone, FT-14 and FT-7 exhibited a ~3 fold decrease while FT-13, FT-18, and FT-12 showed much larger ~10- to 20-fold decreases in the expression levels of these two 14q32 miRNAs (Figure 1D). The qRT-PCR results for 14q32 miRNA correlation also showed high correlation (R^2^ = 0.99) similar to the correlation observed by microarray (R^2^ =0.95).

### Correlations between 14q32 miRNA and mRNA expression patterns predict association between decreased 14q32 miRNA and human osteosarcoma metastasis

**Figure 2:**
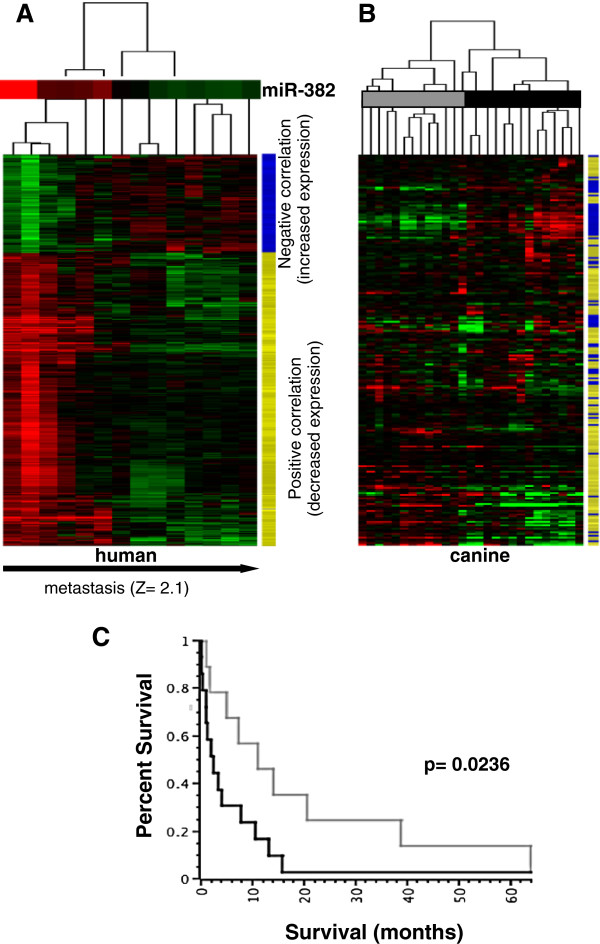
**Prognostic significance of genes correlated with 14q32 miRNAs in canine osteosarcoma.** (**A**) Unsupervised clustering of human mRNAs (n = 385) that shows high-level direct correlations to miR-382 for two normal bone tissues and 11 osteosarcoma tumors, for which we have both mRNA profiles and miRNA profiles. A positive correlation to miR-382 is shown in yellow (n = 288), and a negative correlation to miR-382 is shown in blue (n = 97), at the right of the heatmap. Directional Functional enrichment analyses (Ingenuity Pathways Analyses) shows that correlating transcripts are enriched in transcripts involved in metastasis, and direction of change is consistent with increased activity of metastatic function in tumors with decreased levels of 14q32 miRNA member, miR-382. Identities of positive and negatively correlating mRNA are provided in Additional file 6: Table S5 and Additional file 7: Table S6. (**B**) Unsupervised hierarchical clustering heatmap of canine mRNA found in 26 canine osteosarcoma-derived samples that correspond to human mRNAs correlating to *miR-382*. The yellow and blue bars represent correlations to miR-382 (positive = yellow or negative = blue) observed in the human data indicating that the direction of change shows similar trends between human and canine data.(**C**) Kaplan-Meier survival curves generated using the groups shown in 2B. Survival times are significantly different between the two groups of tumors (p-value 0.02).

To further understand the role of the 14q32 miRNAs in human osteosarcoma, we profiled the mRNA transcript levels of tumor samples for which we also had miRNA profiles. We then determined mRNA that positively and negatively correlated to miR-382. Since all the tested 14q32 miRNAs expression levels were highly correlated on a sample-by-sample basis, we selected miR-382, as a representative of 14q32 miRNAs. The results of this analysis in human osteosarcoma samples uncovered 288 and 97 genes that were positively (Additional file 6: Table S5) and negatively (Additional file 7: Table S6) correlated, respectively, to miR-382 expression (R^2^ > 0.7). All of these 385 genes and their direction of change in relation to miR-382 are shown as a heatmap in Figure 2A. Ingenuity Pathway Analysis (IPA) showed that genes involved in the functional category ‘metastasis’ were enriched in this signature (Enrichment p-value < 0.001 after Benjamini Hochberg multiple testing correction). Furthermore, genes of this signature in the functional category ‘metastasis’ were predicted to have increased activity (Regulation Z-score 2.123) in osteosarcoma tumor samples that showed the lowest levels of 14q32 miRNAs on the basis of the direction of change of the correlated genes. This provides a potential molecular rationale for the observation of metastasis being present in the samples with the largest decreases in 14q32 miRNA transcript levels.

### Gene expression levels correlated with 14q32 miRNAs also predict poor survival in canine osteosarcoma

As a next step in comparative assessment, we mapped the human osteosarcoma genes that positively and negatively correlated with miRNA-382 (Figure 2A, direction of change marked in yellow and blue) to orthologous canine genes that were included in the Affymetrix canine 2.0 platform (Figure 2B). Mapping of the human to canine genes provided 219 canine genes, which we then used to perform unsupervised hierarchical clustering of the 26 canine osteosarcoma samples. These 219 genes segregated the canine osteosarcoma samples into two distinct branches. Kaplan-Meier analysis using death as an outcome determined that there was a statistically significant (p < 0.05) difference in survival between dogs in these two distinct branches (Figure 2C). Dogs in which miR-382-positively correlated mRNAs were expressed at lower levels and in which miR-382-negatively correlated mRNAs were expressed at higher levels had significantly shorter survival times than the other group of dogs. This result shows that genes that correlate with miR-382 are capable of showing survival trends in dogs with osteosarcoma that are consistent with our hypothesis in humans, thus independently supporting a prognostic role for the 14q32 miRNA transcript level in osteosarcoma.

### Expression levels of 14q32 miRNAs is inversely correlated to metastasis in human osteosarcoma patients

Because metastasis genes are enriched in miR-382 correlated gene signature in canine osteosarcoma and miR-382 expression levels showed potential prognostic significance (ability to predict poor outcome) in the canine osteosarcoma cohort, we tested whether expression levels of 14q32 miRNAs in human showed similar association with outcomes. We analyzed the miRNA profiles obtained from the initial cohort of human osteosarcoma tumor tissue samples. Analysis revealed an interesting inverse relationship: the osteosarcoma samples with the lowest levels of 14q32 miRNAs were from patients that developed metastatic tumors (Figure 1B-D and 2A) This human osteosarcoma sample cohort was incompletely annotated (no survival data available), but a Mann Whitney test showed that a potential association was present between levels of 14q32 expression levels and metastasis (p-value 0.1357).

### Independent validation that 14q32 miRNAs are prognostically significant in human osteosarcoma

**Figure 3:**
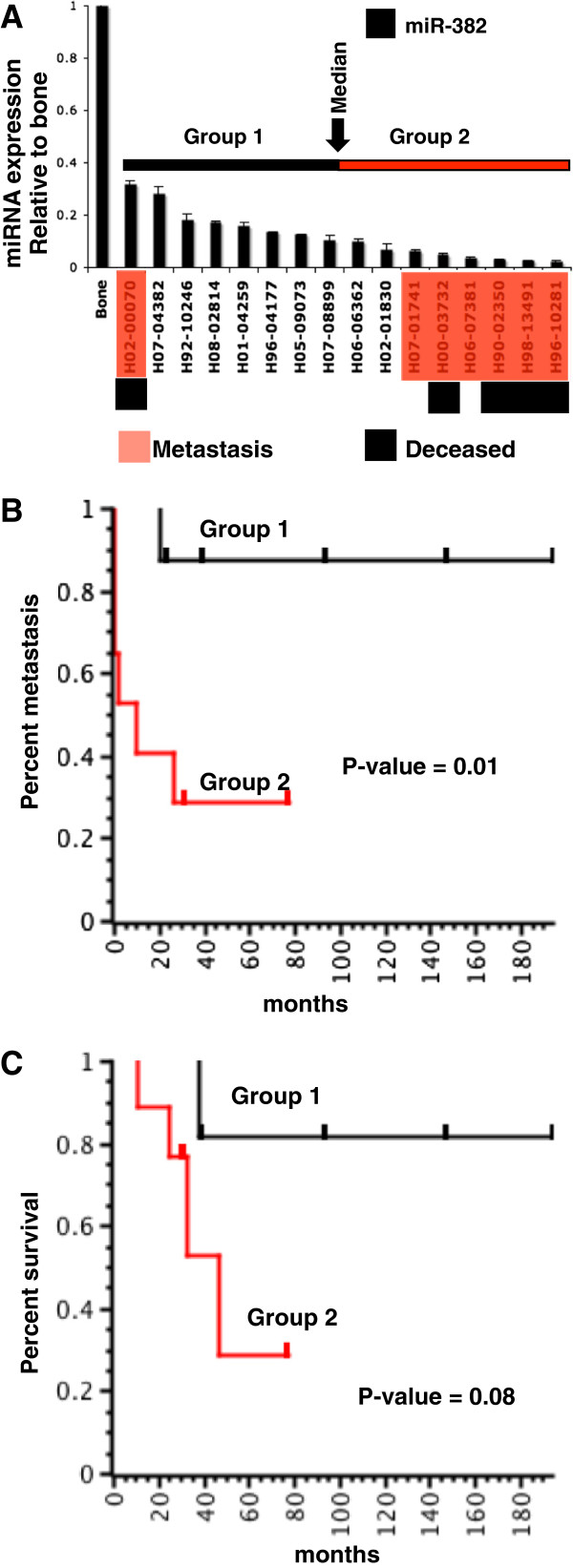
**14q32 miRNA expression level, metastasis and outcome in human osteosarcoma.** (**A**) miR-382 expression levels determined by qRT-PCR in osteosarcoma primary tumor samples with clinical follow-up information. The miRNA expression levels were normalized relative to normal bone tissues. Osteosarcoma samples were ranked based on expression levels of miR-382 from highest to lowest. Primary osteosarcoma patient samples that later developed metastases are highlighted in red and samples from patients who died due to disease are shown in black. Samples were grouped based on whether they were above or below the median expression level. (**B**) Kaplan-Meier analysis of metastasis in osteosarcoma patients based on miR-382 expression. Group 1 and Group 2 are significantly different (p-value 0.01). Patients with lowest levels of miR-382 expression showed increased likelihood of metastasis. (**C**) Kaplan-Meier analysis of survival in osteosarcoma patients based on miR-382 expression. Group 1 and Group 2 are different (p-value 0.08). Patients with lowest levels of miR-382 expression showed decreased likelihood of survival.

To further examine the association between low 14q32 miRNA levels in osteosarcoma tumors and clinical outcomes, we tested a representative 14q32 miRNA (miR-382) in an independent set of primary tumor samples from 16 human osteosarcoma patients that had more robust clinical follow-up annotation (Additional file 3: Table S3). As determined by qRT-PCR, all of the osteosarcoma tumor tissue samples in this independent set showed lower levels of miR-382 compared to normal bone tissues (Figure 3A). As we noticed in the first human osteosarcoma dataset, the lowest levels of miR-382 were found in primary osteosarcoma tumors from patients where metastases and/or death were later observed as follow-up. We further noticed that there was a negative correlation between the levels of miR-382 expression and patient survival (Figure 3A and see Additional file 3: Table S3). To test whether lower levels of 14q32 were significantly associated with metastasis, we used the Mann Whitney test. This test allowed us to reject the null hypothesis that there was no association between 14q32 level and metastasis (p < 0.01).

Further, to examine the prognostic utility of the 14q32 miRNA level, we split the cohort into two groups based on whether the miR-382 level was above the median value (Group 1) or below the median value (Group 2). We compared the two groups using Kaplan-Meier analyses using both metastasis and death as outcomes (Figure 3B and 3C). Human osteosarcoma tumor samples that had the lowest levels of miR-382 were at higher risk for metastasis (p-value 0.01) and showed a trend for death (p-value 0.08) than patients with moderate miR-382 expression independently confirming the observation that 14q32 miRNAs are a potential prognostic tool for osteosarcoma.

### Human 14q32 miRNAs are conserved and prognostically significant in canine osteosarcoma

**Figure 4:**
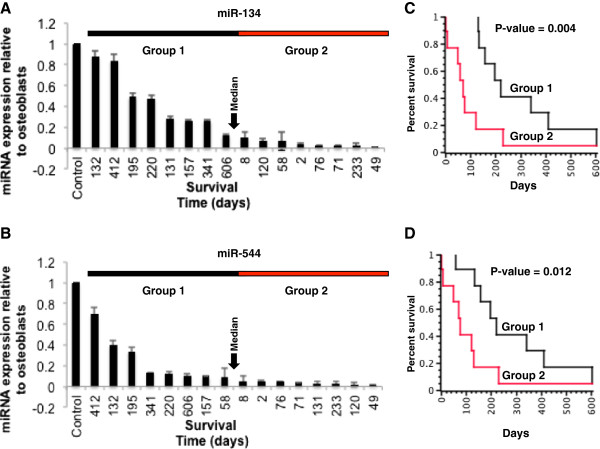
**14q32 orthologous region transcript levels and outcome in canine osteosarcoma.** (**A**) miR-134 and B) miR-544 expression levels determined by qRT-PCR in canine osteosarcoma primary tumor samples with clinical follow-up information. The miRNA expression levels were normalized relative to reactive canine osteoblasts. Osteosarcoma samples were ranked based on expression levels of miR-134 from highest to lowest. Samples were grouped based on whether they were above or below the median expression levels. (**C** and **D**) Kaplan-Meier analysis of survival in osteosarcoma patients based on miR-134 and miR-544 expression. Survival of dogs with osteosarcoma in Group 1 and Group 2 are significantly different (miR-134 p-value 0.004, miR-544 p-value 0.01). Dogs with lowest levels of miR-134 or miR-544 expression showed decreased likelihood of survival.

We examined the orthologous 14q32 locus miRNAs and genes within the canine genome using the Entrez gene database and were able to find them on CFA 8 (Additional file 5: Figure S1). Similar to human 14q32 locus, canine orthologous miRNAs were also found in the syntenic region between genes *DLK1* and *DIO3*. The order of the miRNA is retained in the canine genome although differences in spacing were observed between human and canine versions. Because we had observed systematic decreases in human 14q32 miRNAs and the magnitude of the decrease showed potential prognostic utility, we hypothesized that the orthologous canine miRNAs on CFA 8 would also show decreases in transcript level in canine osteosarcoma tumors and that the magnitude of this decrease would show prognostic utility. To address this, we determined the levels of miR-544 and miR-134 (based on identical mature miRNA sequences) in 16 canine osteosarcoma tumors from which RNA was available by qRT-PCR (Figure 4A). In all 16 cases, expression decreases were observed in the miRNA relative to reactive canine osteoblasts. As observed in human osteosarcoma the levels of miR-544 and miR-134 were directly correlated (R^2^ = 0.88)

The canine osteosarcoma samples that were analyzed by qRT-PCR were derived from dogs with overall survival times ranging from 2 to 606 days with a median survival of 131.5 days (Additional file 4: Table S4). The canine cohort was separated into two groups using the same method used for the human tumor samples. Group 1 was composed of tumor samples with miR-134 (Figure 4A) and miR-544 (Figure 4B) expression levels above the median survival days and Group 2 was composed of tumor samples below the median, and these two groups were interrogated via Kaplan-Meier survival analysis (Figure 4D). Dogs with tumor with lower levels of miR-134 had higher risk for death (p-value 0.004) than dogs with tumors with higher levels of miR-134 (Figure 4C). Similar results were obtained using miR-544 expression levels (p-value 0.012, Figure 4D). Together, these results indicate that the 14q32 miRNA expression levels have prognostic utility in osteosarcoma, a disease that shows conserved features between humans and dogs.

## Discussion

Molecular markers to identify osteosarcoma patients at risk for metastatic progression are critical to adopting suitable treatment options. Recently, several molecular markers based on gene expression in tumor tissues have been identified in osteosarcoma. Higher levels of oncogene YY1 expression in primary sites of osteosarcoma are associated with metastasis and poor clinical outcomes [18]. Similarly, overexpression of markers such as cancer-testis antigens, Cystein Rich Protein (CYR61) and Melanoma Antigen Family A (MAGEA) are implicated in predicting tumor progression and prognosis in osteosarcoma [19,20]. Additionally, the presence of tumor-infiltrating macrophages is associated with metastasis suppression in high-grade osteosarcoma [21]. These studies show that the development of biomarkers for tumor progression and outcomes have clinical significance and may help develop patient specific treatment strategies. Recently, miRNAs from tumor tissues have been proposed for use in the diagnosis, classification and the prognosis of various cancers [22-26].

The cluster of miRNAs at the 14q32 locus show decreased levels in human osteosarcoma [10]. miRNA profiling of murine osteosarcoma tumors also show decreases in the orthologuos region of chromosome 12 relative to the levels observed in normal bone tissues (personal communication). Here, we report decreases in miRNA in the orthologous region of CFA 8 cluster members in canine osteosarcoma tumors. This is consistent with the presence of a commonly conserved mechanism of tumorigenesis involving the orthologous 14q32 locus associated genes and miRNAs between these species. Thus, the cross species comparison of human and canine expression profiles provides an opportunity to define the existence of evolutionarily conserved molecular signatures and to determine whether those signatures influence the outcome of our 3 patient cohorts (2 human and 1 canine).

In both the human and canine osteosarcoma cases the 14q32 miRNAs and its orthologs appear to be regulated by a common mechanism, based on the high levels of expression correlation observed between the transcript levels of each member. Analyses of the Sarcoma MicroRNA Expression Database (SMED) [9] data reveal that 14q32 miRNA, as a group, are lost in a subset of GIST (3/14) and MFH (5/29). We conclude from this that the level of 14q32 miRNAs in general can be estimated from the measurement of a single member of the family and that measurement of the majority of the 14q32 miRNAs would provide highly similar data. Polycistronic regulation of these miRNAs has previously been proposed as a regulatory mechanism active at this locus and our data supports this concept [17].

In an initial set of human osteosarcoma tumors miRNA microarray profiling data we observed that lower levels of 14q32 miRNA were associated with metastases. We further validated this by qRT-PCR for both miR-382 and miR-154 for osteosarcoma tumors from this initial dataset. We also showed that the lowest levels of miR-382 were significantly associated with metastasis and significantly worse survival in a second independent set of human primary osteosarcoma tumors where full clinical follow-up information was available. Taken together, these results suggest that the level of miR-382 may have prognostic utility for predicting the likelihood of developing metastatic tumors and disease outcomes. This observation is supported by three independent dataset from two different species. While the association between reduced expression of 14q32 miRNAs and overall survival in humans represented a trend, in dogs, the magnitudes of miR-134 and miR-154 reduction were negative predictors of survival. This is likely due to the fact that, unlike humans (and especially children) where preserving life is the guiding principle, companion animal patients are treated with quality of life as the guiding principle. Therefore, metastatic disease is seldom treated aggressively and recurrence often leads to a decision of humane euthanasia, creating little difference between the disease-free interval and overall survival [27]. It is prudent to note, however, that osteosarcoma is a highly heterogeneous disease and the sample sizes of the cohorts used in this study were relatively small. Thus, our conclusion will require additional validation in a larger cohort of osteosarcoma patients with clinical follow-up.

We have previously shown that decreases in 14q32 miRNA levels stabilize cMYC expression in osteosarcoma and subsequently increase the expression of oncogenic miR-17-92 miRNAs [10]. Deregulations in 14q32 miRNA-cMYC network is also associated with increased proliferation and apoptotic escape in osteosarcoma [10]. The prognostic relevance of decreasing 14q32 miRNA levels may be explained 1) by increased activation of the cMYC/miR-17-92 network leading to poor outcomes 2) via regulation of an alternative cancer signaling pathway(s) either directly or indirectly or 3) as a biomarker whereby decreased levels of 14q32 miRNAs are observed in osteosarcoma cases with poor outcomes.

Genes involved in metastasis are significantly enriched in transcripts correlated to 14q32 miRNAs and the direction of change indicates that metastasis function is also significantly increased (Additional file 6: Table S5 and Additional file 7: Table S6). CDK5 and TWIST1 have been reported to increase metastasis [28,29] and are upregulated in osteosarcoma tumors with low levels of 14q32 miRNAs. IFNB1 [30], TEK [31] and COL18A1 [32,33] have been reported to decrease metastasis and are downregulated in tumors with low levels of 14q32 miRNAs. We also noticed that *TK1* is negatively correlated to 14q32 miRNA levels. *TK1* is involved in the catalysis of phosphorylation of thymidine to deoxythymidine monophosphate and expressed at high levels in proliferating cells and appears to correlate with high risk in multiple cancer types [34-36]. This suggests that genes correlated with 14q32 miRNA expression patterns may provides molecular rational for the observed association between low levels of 14q32 and metastasis and poor survival outcomes and can be further investigated as markers of prognosis.

We have previously demonstrated that comparative studies of human and canine osteosarcoma patients allow study of evolutionary conserved mechanisms of tumorigenesis associated with outcomes [6]. A majority of the dogs evaluated in this study (21/25 in the correlating miRNA analysis and 12/16 in the qRT-PCR analysis) were treated with standard of care, which consists of amputation of the limb to remove the primary tumor followed by with adjuvant chemotherapy. Predictably, treatment generally was associated with increased survival, but the negative correlation with miR-134 and miR-154 was not driven by the type of treatment that the dogs received, and in fact, the data suggest that the dogs that received palliative care and amputation (2, 8 and 58 days survival) would have been reasonable candidates for more prolonged survival with standard of care treatment.

## Conclusions

We have identified and confirmed an association between metastasis and 14q32 miRNA expression levels in human osteosarcoma. Further, we have shown that the transcript decreases and prognostic significance for 14q32 miRNAs are conserved in canine osteosarcoma. Based on these integrative, comparative, multi-species analyses, we conclude that larger decreases in 14q32 miRNA expression levels are associated with an increased likelihood of metastases and poor outcomes in osteosarcoma patients.

## Competing interests

The authors have no Conflict of Interest.

## Authors’ contribution

ALS, VT, MCS, JFM, SS conceived and designed the experiments; ALS, VT, MCS, AC, CJ, PCWH, JFM, SS Contributed reagents/materials/analysis tools; ALS, VT, MCS, performed the experiments and analysis; ALS, VT, MCS, JFM, SS wrote the paper. All authors read and approved the final manuscript.

## Supplementary Material

Additional file 1: Table S1 Supplementary Table 1: Demographic and clinical information of human OS patient samples. Click here for file

Additional file 2: Table S2 Demographic and clinical information of normal bone sampels used in the study. Click here for file

Additional file 3: Table S3 Human OS patient sample information and clinical follow-up. Click here for file

Additional file 4: Table S4 Clinical information of canine OS cases. Click here for file

Additional file 5: Figure S1 Homology between human 14q32 miRNAs and canine chr 8:72.3 Mb miRNAs locus. Data were obtained from Entrez gene database (April 15 2012). (A-B) Conservation at the gene level. (C-D) Conservation at the miRNA level. While the order is highly maintained, only certain miRNAs are shown for the purpose of illustration. The position of miR-379 was used to orient the orthologous canine region of the genome to the human 14q32 locus. Click here for file

Additional file 6: Table S5 Differentially expressed mRNA in osteosarcoma positively correlated to 14q32 encoded miR-382. Click here for file

Additional file 7: Table S6 Differentially expressed mRNA in osteosarcoma negativelly correlated to 14q32 encoded miR-382. Click here for file

Additional file 8: Table S7 14q32 subnetwork pairwise correlations. Click here for file
