# Supplementary material for: MicroRNAs at the human 14q32 locus have prognostic significance in osteosarcoma
Source: Orphanet J Rare Dis. 2013 Jan 11;8:7. doi: 10.1186/1750-1172-8-7 (PMC3566973; doi:10.1186/1750-1172-8-7)
Supplement: Additional file 5: Figure S1 — Homology between human 14q32 miRNAs and canine chr 8:72.3 Mb miRNAs locus. Data were obtained from Entrez gene database (April 15 2012). (A-B) Conservation at the gene level. (C-D) Conservation at the miRNA level. While the order is highly maintained, only certain miRNAs are shown for the purpose of illustration. The position of miR-379 was used to orient the orthologous canine region of the genome to the human 14q32 locus. [file 1750-1172-8-7-S5.ppt]

## Slide 1
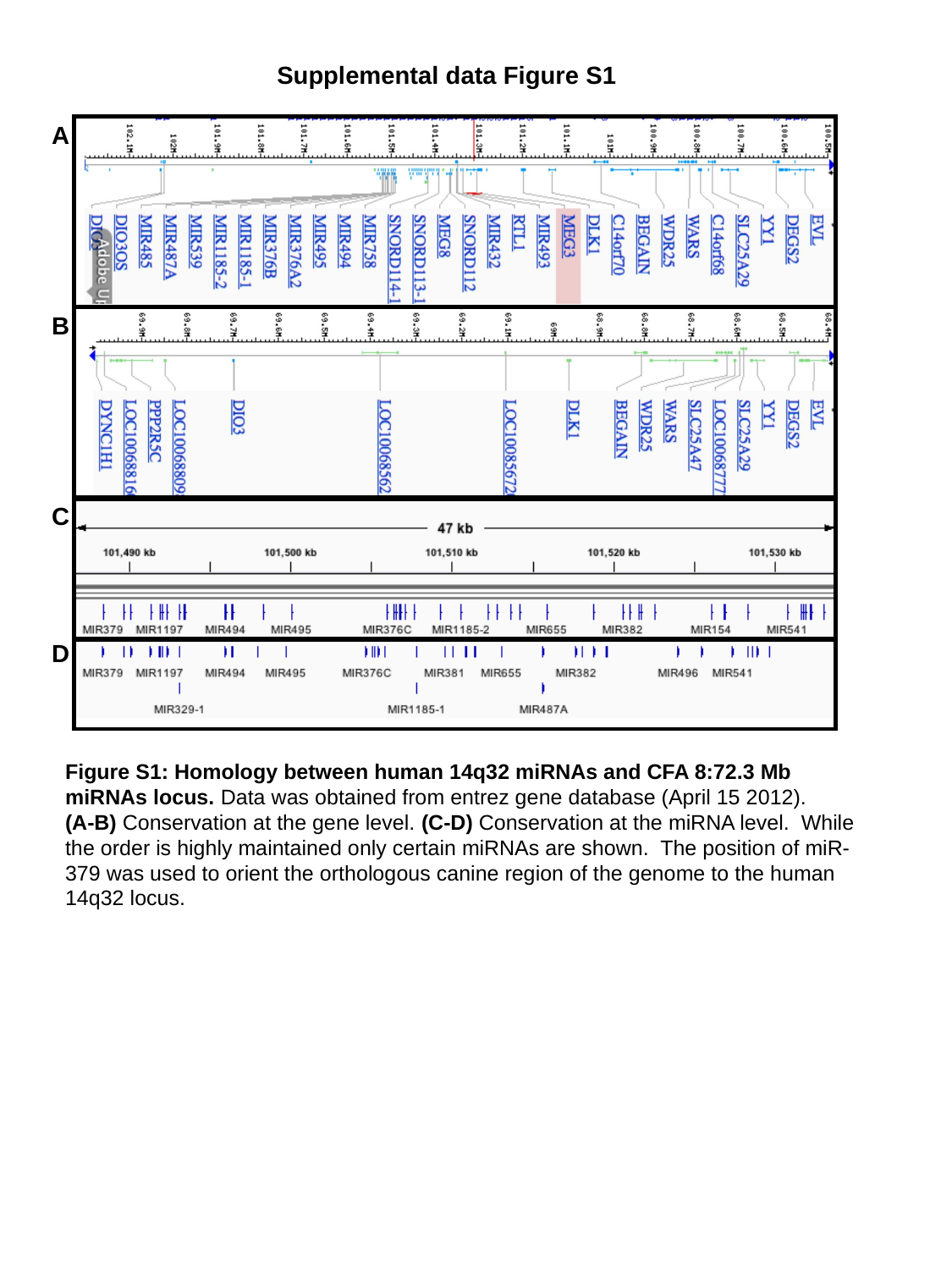

Supplemental data Figure S1
A
B
C
D
Figure S1: Homology between human 14q32 miRNAs and CFA 8:72.3 Mb miRNAs locus. Data was obtained from entrez gene database (April 15 2012).
(A-B) Conservation at the gene level. (C-D) Conservation at the miRNA level. While the order is highly maintained only certain miRNAs are shown. The position of miR-379 was used to orient the orthologous canine region of the genome to the human 14q32 locus.
